# HIV-2 genomic RNA accumulates in stress granules in the absence of active translation

**DOI:** 10.1093/nar/gku1017

**Published:** 2014-10-28

**Authors:** Ricardo Soto-Rifo, Fernando Valiente-Echeverria, Paulina S. Rubilar, Francisco Garcia-de-Gracia, Emiliano P. Ricci, Taran Limousin, Didier Décimo, Andrew J. Mouland, Théophile Ohlmann

**Affiliations:** 1 Programa de Virología, Instituto de Ciencias Biomédicas, Facultad de Medicina, Universidad de Chile, Independencia 8389100, Santiago, Chile; 2 HIV-1 RNA Trafficking Laboratory, Lady Davis Institute at the Jewish General Hospital, Montréal, Québec, H3T 1E2, Canada; 3 Department of Medicine, Division of Experimental Medicine and Department of Microbiology & Immunology, McGill University, Montréal, Quebec, H3A 2B4, Canada; 4 INSERM U1111, CIRI, Lyon, F-69364, France; 5 Ecole Normale Supérieure de Lyon, Lyon, F-69364, France

## Abstract

During the post-transcriptional events of the HIV-2 replication cycle, the full-length unspliced genomic RNA (gRNA) is first used as an mRNA to synthesize Gag and Gag-Pol proteins and then packaged into progeny virions. However, the mechanisms responsible for the coordinate usage of the gRNA during these two mutually exclusive events are poorly understood. Here, we present evidence showing that HIV-2 expression induces stress granule assembly in cultured cells. This contrasts with HIV-1, which interferes with stress granules assembly even upon induced cellular stress. Moreover, we observed that the RNA-binding protein and stress granules assembly factor TIAR associates with the gRNA to form a TIAR-HIV-2 ribonucleoprotein (TH2RNP) complex localizing diffuse in the cytoplasm or aggregated in stress granules. Although the assembly of TH2RNP in stress granules did not require the binding of the Gag protein to the gRNA, we observed that increased levels of Gag promoted both translational arrest and stress granule assembly. Moreover, HIV-2 Gag also localizes to stress granules in the absence of a ‘packageable’ gRNA. Our results indicate that the HIV-2 gRNA is compartmentalized in stress granules in the absence of active translation prior to being selected for packaging by the Gag polyprotein.

## INTRODUCTION

During the late steps of the HIV-2 replication cycle, the full-length, unspliced genomic RNA (hence referred as gRNA) plays a critical role by acting as both an mRNA template for the synthesis of the structural and enzymatic proteins (Gag and Gag-Pol) and as the viral genome that is selected and packaged into newly produced viral particles (1–3). The 5′-untranslated region (5′-UTR) is ordered in highly structured and conserved RNA motifs that contribute to the regulation of gRNA translation and packaging and, as a consequence, Gag synthesis is in close relationship with the viral assembly process (2–4). The molecular mechanisms that control ribosome recruitment onto the HIV-2 gRNA have been elucidated over the last years (4). Despite the presence of an m^7^GpppG cap structure, the HIV-2 TAR RNA motif strongly interferes with ribosome recruitment at the 5′ end resulting in low translational rates and limited levels of Gag synthesis compared to the mechanism used by HIV-1 (5). This could explain why HIV-2 Gag is mostly produced by internal initiation of translation (6–9) and by the use of a 5′-UTR spliced variant of the gRNA that destabilizes TAR (5,10). Interestingly, it has been proposed that this limited production of Gag is required during the HIV-2 assembly process in order to ensure the selection of the gRNA over the rest of viral transcripts that also posses the major packaging signal (11,12). However, how translation and the selective packaging of the gRNA are coordinated to allow enough levels of Gag synthesis prior to the switch to virus assembly remains largely unknown.

The assembly of cytoplasmic mRNA granules such as P-bodies and stress granules is inversely related to active translation (13). While P-bodies are enriched in components of the mRNA decay machinery and are constitutively present in cells, stress granules correspond to highly dynamic structures containing mRNAs that are stalled at the translation initiation stage in response to diverse cellular stresses (13). Given the critical post-transcriptional control occurring in these mRNA granules, it is not surprising that many RNA viruses manipulate stress granules and P-bodies or some of their components during different steps of their replication cycles (14,15).

Our recent data showed that HIV-1 has the ability to interfere with stress granule assembly (16,17). Indeed, we observed that the HIV-1 gRNA rather associates with the cellular protein Staufen1 to form a highly specific viral mRNP in which the viral protein Gag is recruited (14,16). This viral mRNP, called Staufen1 HIV-1-dependent RNP (SHRNP), was found to be compositionally different than stress granules as they lacked classical markers such as TIAR, TIA-1, G3BP1 or PABP (16). Interestingly, the assembly of SHRNPs imposed a blockade to stress granule assembly, which was associated to processes governing HIV-1 gRNA encapsidation (14,16). Moreover, the deltaretrovirus Human T-lymphotropic virus-1 (HTLV-I) has also been shown to interfere with stress granule assembly, suggesting that this property could be a common feature of the retrovirus family (14,18). However, whether the ability to interfere with stress granule assembly is conserved in HIV-2 remains to be determined.

In this work, we used cell imaging coupled to functional assays in order to examine the cytoplasmic post-transcriptional regulation of the HIV-2 gRNA. This led to the identification of the RNA-binding protein and stress granule assembly factor TIAR as a host factor required for HIV-2 replication. Interestingly, we report that the HIV-2 gRNA recruits TIAR to form a gRNA/TIAR (TIAR-HIV-2 ribonucleoprotein complex, TH2RNP) complex that localizes diffusely in the cytoplasm or in stress granules in response to eIF2α phosphorylation. These observations suggested that stress granule assembly is required for viral replication. Indeed, we observed a correlation between the accumulation of the gRNA in stress granules and the translational arrest occurring in response to increased levels of Gag. Taken together, our results suggest that the HIV-2 gRNA found in stress granules corresponds to a novel post-translational intermediate likely involved in a switch between a translationally active state toward a packaging prone one.

## MATERIALS AND METHODS

### DNA constructs

pRod10 and pRod10-Renilla were previously described (5,19). The HIV-2 ROD-derived pSVR mutant vectors were previously described (11,12,20). The infectious HIV-1 molecular clone NL4–3 was described previously (21). cDNA sequences for G3BP1, TIAR and FXR1 were obtained by reverse transcriptase-polymerase chain reaction (RT-PCR) using total RNA extracted from either HeLa cells or umbilical cordon cells. Digested and purified RT-PCR products for G3BP1, TIAR and FXR1 were cloned into the EcoRI or MluI sites of pEGFP-C1 (Clontech). The EGFP and Renilla ORF were cloned into the EcoRI-NotI sites of the pCI-HA vector previously described (22). The DCP1-EGFP vector was previously described (23).

### Cell culture and DNA transfection

HeLa cells were maintained in Dulbecco's modified Eagle's medium growth media (Gibco, BRL) supplemented with 10% fetal calf serum at 37°C and 5% CO_2_. DNA transfection was carried out using JetPEI™ (PolyPlus Transfection) following supplier's indications. Cells or supernatants were recovered at 24 h post transfection and used either for Renilla activity, RNA extraction and RT-qPCR or western blot as indicated on the figures. To induce stress, Pateamine A (PatA, from Dr Jerry Pelletier, McGill University, Canada) and sodium Arsenite (Sigma-Aldrich) were used. PatA was diluted in media to a final concentration of 300 nM and applied to cells for 1 h prior to collection. Arsenite was diluted to a final concentration of 0.5 mM and applied to the cells for 45 min prior to collection.

### Antibodies

Mouse anti-p24 was obtained from the National Institutes of Health AIDS Reference and Reagent Program. Anti-eIF4A (provided by Dr Simon Morley, University of Sussex, UK); anti-G3BP1 (provided by Dr Imed Gallouzi, McGill University, Canada); anti-TIAR (Santa Cruz Biotechnology); anti- eIF2α-P (Abcam); anti-eIF4GI (provided by Dr Nahum Sonenberg, McGill University, Canada); anti-eIF3b (Santa Cruz Biotechnology) and AlexaFluor fluorophore conjugates (Life Technologies).

### RNA immunoprecipitation

Transfected cells were washed with phosphate buffered saline (PBS), recovered by centrifugation at 2500 rpm for 10 min at 4°C and resuspended in 400 μl of hypotonic gentle lysis buffer (10 mM Tris-HCl pH = 7.5; 10 mM NaCl, 10 mM EDTA; 0.5% Triton X-100; 2 mM Ribonucleoside Vanadyl Complex (VRC) and protease inhibitors cocktail). Cells were incubated for 10 min on ice, NaCl was adjusted to 150 mM and the lysate was cleared by centrifugation at 10 000 rpm for 10 min at 4°C. The supernatant was incubated with 10 μg of a mouse anti-HA antibody (Covance) or 10 μg of mouse IgG1 (Millipore) for 3–4 h at 4°C, 10 rpm rotation. Protein–antibody complexes were recovered with 50 μl of Dynabeads^®^ Protein G (Life Technologies) during 1 h at 4°C, 10 rpm rotation and recovered complexes were washed extensively with NET-2 buffer (50 mM Tris-HCl pH = 7.5; 150 mM NaCl; 0.05% Nonidet P40). Bound material was split in two and was either resuspended in Sodium dodecyl sulfate (SDS)-loading buffer for western blot analyses or treated with Proteinase K during 1 h at 52°C to then be subjected to RNA extraction as described below. The presence of HIV-2 gRNA in TIAR-bound RNAs was verified by conventional PCR.

### RNA extraction and RT-qPCR

RNA extraction and RT-qPCR were performed as previously described (24). Briefly, HeLa cells transfected with Renilla-based DNAs were washed four times with PBS and lysed with 200 μl of RLNa buffer (10 mM Tris-HCl [pH 8.0], 10 mM NaCl, 3 mM MgCl2, 1 mM DTT, 0.5% NP40 and 15 U/ml of RNaseOUT (Invitrogen Co.)). When necessary, whole cell extracts were centrifuged at 13 000 rpm during 4 min to pellet nuclei and obtain the cytoplasmic fraction. Whole cell extracts (for total RNAs) or cytoplasmic fractions were recovered and RNA extraction was carried out by adding 1 ml of TRIzol^®^ Reagent (Invitrogen Co.) as indicated by the manufacturer. Extracted cytoplasmic RNAs (200 ng) were reverse-transcribed using the qScript™ Flex cDNA kit (Quanta Biosciences). For quantitative PCR, a 20 μl reaction mix was prepared with 5 μl of template cDNAs (previously diluted to 1/10), 10 μl of MESA green SYBR qPCR MasterMix (Eurogentec), 0.2 μM of sense and antisense primers and subjected to amplification using a fluorescence thermocycler (Applied Biosystems 7000 Real-time PCR, Foster City, CA, USA). The housekeeping gene GAPDH was amplified in parallel to serve as a control reference. Relative copy numbers of Renilla luciferase cDNAs were compared to GAPDH using x^−ΔCt^ (where x corresponds to the experimentally calculated amplification efficiency of each primer couple).

### Renilla activity

Renilla activity was measured using the Renilla Luciferase Assay System (Promega Co, Madison, WI, USA) in a Veritas Luminometer (Turner Biosystems) as previously described (25,26).

### Fluorescence *in situ* hybridization (FISH) and confocal microscopy

**Figure 1.**
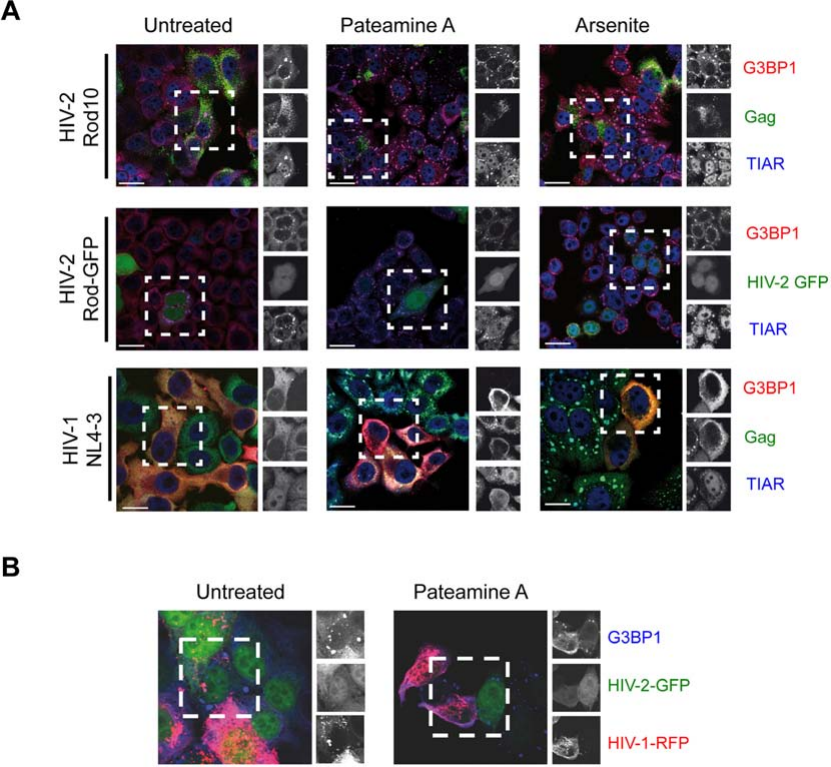
HIV-2 induces cytoplasmic granules. (**A**) HeLa cells were transfected with HIV-2 Rod10, HIV-2 Rod-GFP or pNL4–3 and were untreated (left panels) or exposed to Pateamine A (middle panel) for 1 h or sodium Arsenite (right panel) for 45 min. Cells were stained for G3BP1, TIAR and Gag. Scale bar 10 μm. (**B**) HeLa cells co-transfected with HIV-2 Rod-GFP and pNL4–3-RFP were maintained untreated or subjected to stress with Pateamine A and stained for G3BP1. Scale bar 10 μm. Images are representative from three separate experiments with *n* = 100 cells analyzed each.

**Figure 2.**
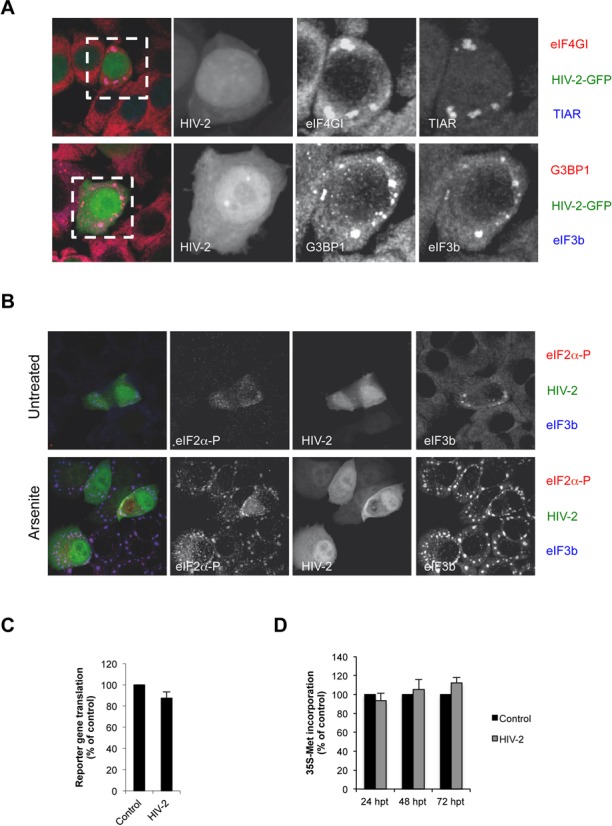
HIV-2-induced granules are *bona fide* stress granules. (**A**) HeLa cells were transfected with HIV-2 Rod-GFP and stained for TIAR and eIF4GI (upper panels) or G3BP1 and eIF3b (lower panels) and analyzed by confocal microscopy as described in Materials and Methods. (**B**) HeLa cells were transfected with HIV-2 Rod-GFP and stained for eIF2α-P (Ser51) and eIF3b in the absence (untreated) or presence of Sodium Arsenite for 45 min (Arsenite) and analyzed by confocal microscopy as described in Materials and Methods. (**C**) HeLa cells were transfected with 0.3 μg of a vector expressing Renilla luciferase. Renilla activity and Renilla mRNA levels were determined 24 h post transfection (hpt) under the following experimental conditions: co-transfection with 1 μg of pCI-HA-EGFP control vector or co-transfection with 1 μg of HIV-2 (pRod10) proviral DNA as indicated on the figure. Results are presented as Renilla activity/mRNA levels normalized to control (arbitrary set to 100%) and correspond to the main +/− SD of three independent duplicate experiments. (**D**) HeLa cells transfected with a control vector or with HIV-2 Rod10 were pulsed with ^35^S-methionine at different time points as described in Materials and Methods. Results are presented as percentage of the control (arbitrary set to 100%) and correspond to the main +/− SD of three independent duplicate experiments.

**Figure 3.**
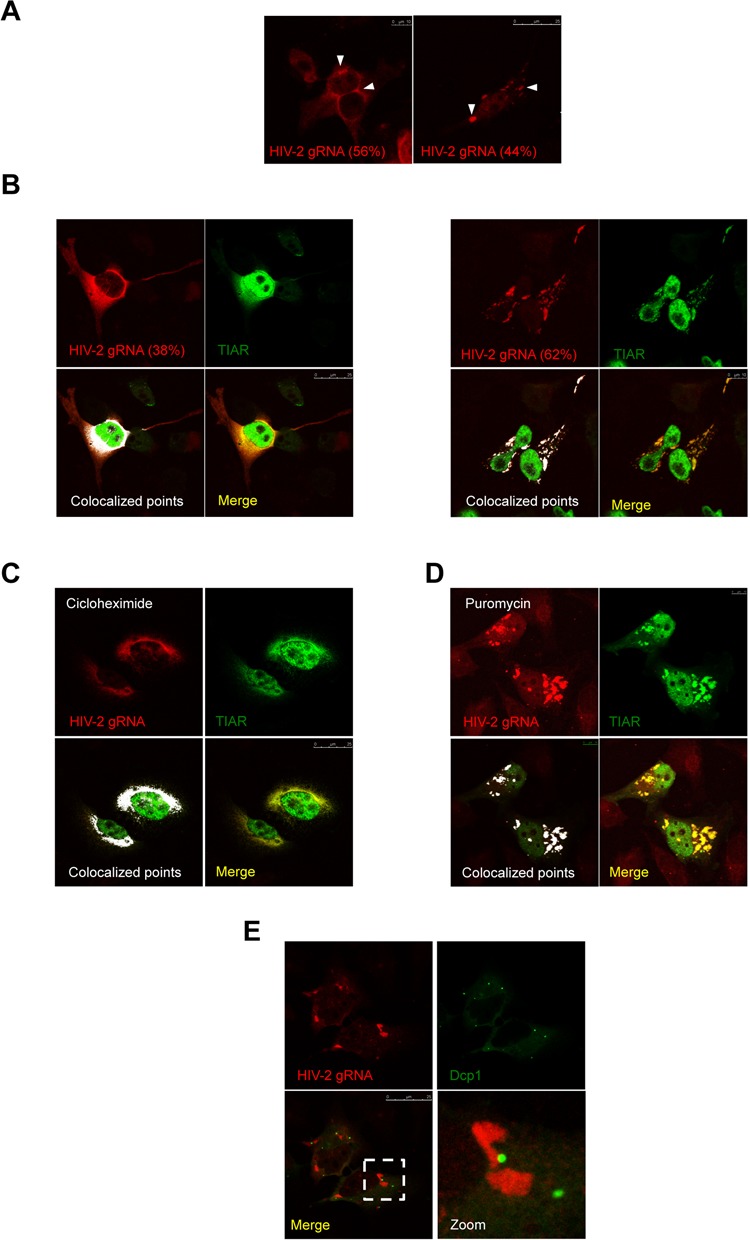
HIV-2 gRNA localizes in stress granules. (**A**) HeLa cells transfected with 0.5 μg of HIV-2 proviral DNA (pRod10) were subjected to RNA FISH and spectral confocal microscopy as described in Materials and Methods. HIV-2 gRNA was observed diffuse in the cytoplasm (left image, scale bar 10 μm) or in large cytoplasmic granules (see arrows in right image, scale bar 25 μm). The percentage of each localization pattern is indicated and images are representative of *n* = 187 cells. (**B**) HeLa cells were co-transfected with 0.5 μg of HIV-2 proviral DNA (pRod10) and 0.25 μg of pEGFP-TIAR and subjected to RNA FISH and spectral confocal microscopy as described in Materials and Methods. HIV-2 gRNA and TIAR co-localize diffuse in the cytoplasm (left images, scale bar 25 μm) or in large cytoplasmic granules (right images, scale bar 10 μm). Images are representative of *n* = 266 cells. **(C)** HeLa cells were co-transfected with 0.5 μg of HIV-2 proviral DNA (pRod10) and 0.25 μg of pEGFP-TIAR and treated with cycloheximide (50 μg/ml) during 2 h prior fixation. Cells were subsequently subjected to RNA FISH and spectral confocal microscopy as described in Materials and Methods. Scale bar 25 μm. **(D)** HeLa cells were co-transfected with 0.5 μg of HIV-2 proviral DNA (pRod10) and 0.25 μg of pEGFP-TIAR and treated with puromycin (0.25 mg/ml) during 4 h prior fixation. Cells were subsequently subjected to RNA FISH and spectral confocal microscopy as described in Materials and Methods. Scale bar 10 μm. **(E)** HeLa cells were co-transfected with 0.5 μg of HIV-2 proviral DNA (pRod10) and 0.25 μg of pEGFP-Dcp1 and subjected to RNA FISH and spectral confocal microscopy as described in Materials and Methods. Scale bar 25 μm.

**Figure 4.**
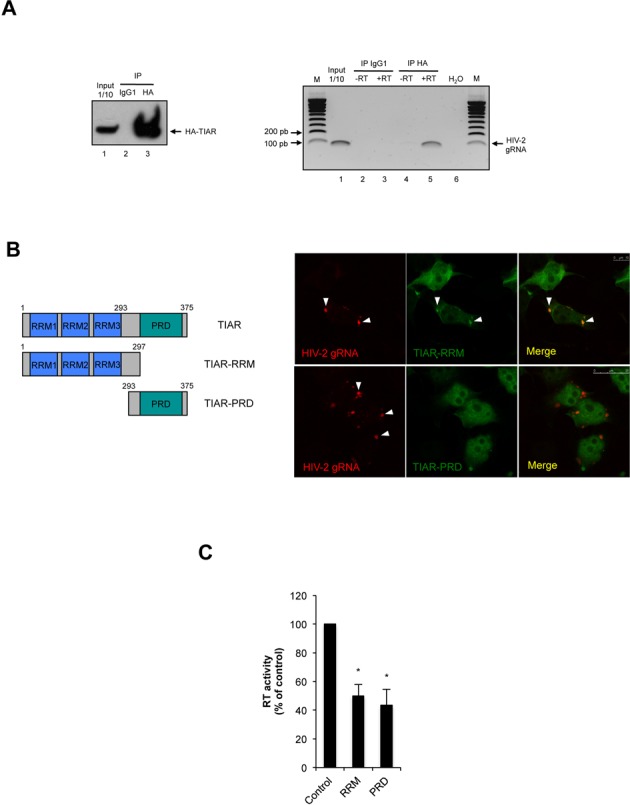
TIAR associates with the HIV-2 gRNA. **(A)** HeLa cells were co-transfected with the HIV-2 proviral DNA (pRod10) and the pCIneo-HA-TIAR vector and cytoplasmic extracts were subjected to immunoprecipitation using a mouse anti-HA antibody (mouse IgG1 was used as a control). The immunoprecipitated material was used for RNA extraction and RT-PCR analyses (with or without RT) using primers specific for the HIV-2 gRNA. **(B)** HeLa cells were co-transfected with 0.5 μg of HIV-2 proviral DNA (pRod10) and 0.25 μg of pEGFP-TIAR RRM (upper panels) or pEGFP-TIAR PRD (lower panels) and subjected to RNA FISH and spectral confocal microscopy as described in Materials and Methods. HIV-2 gRNA and TIAR-RRM co-localized in large cytoplasmic granules (upper panel, scale bar 10 μm) but not with TIAR-PRD (lower panel, scale bar 25 μm) as indicated by arrowheads. **(C)** HeLa cells were co-transfected with the HIV-2 proviral DNA (pRod10) and either pEGFP-TIAR RRM or pEGFP-TIAR PRD and cell culture supernatants were used to determine the RT activity as described in Materials and Methods. Results were normalized to the pEGFP control (arbitrary set to 100%) and correspond to the main +/− SD of three independent duplicate experiments. (* correspond to a *P*-value < 0,05).

HeLa cells were cultured in Lab-Tek™ Chamber Slides (Nunc™) and were maintained and transfected with DNA constructs as described above. For RNA FISH, cells were washed twice with 1X PBS and fixed for 10–15 min at room temperature with 4% paraformaldehyde/1X PBS. Cells were washed twice with 1X PBS and permeabilized for 5 min at room temperature with 0.2% Triton X-100/1X PBS. Cells were washed twice with PBS and hybridized overnight at 37°C in 200 μl of hybridization mix (10% dextran sulfate, 2 mM vanadyl-ribonucleoside complex, 0.02% RNase-free BSA, 50% formamide, 300 μg tRNA and 150 ng of a specific Cy5-labeled LNA-modified probe directed against the Pol coding region within the HIV-2 gRNA (5′-GGATTAGTTGGAGGTGCTTCCTCTAGCTGGCCTTCTTTTTCC-3′, LNA nucleotides are underlined). Cells were washed with 2X SSC/50% formamide during 30 min at 37°C and mounted in Moviol. Images presented in Figures 3, 4, 6 and 7 were obtained with a TCS SP5 AOBS Spectral Confocal Microscope (Leica Microsystems) using immersion oil and the 63X lens objective. Images for each fluorophore (EGFP and Cy5) were obtained by sequential scan using two independent channels and correspond to the average of eight frames. Images were merged and recovered using the LAS AF Lite software (Leica Microsystems). Co-localization analyses were carried out using ImageJ. Images from Figures 1 and 2 were obtained as previously described (27). Briefly, cells were washed in PBS before being blocked in 1X blocking solution (Roche). The indicated primary antibodies were applied for 1 h at 37°C, and then washed for 10 min in PBS followed by incubation with secondary antibodies for 1 h. Laser scanning confocal microscopy was performed using a Zeiss LSM5 Pascal confocal microscope equipped with a 63x (1.4 numerical aperture, oil immersion) plan-apochromat objective lens and an Ar/Kr laser. LMS Image Browser (Carl Zeiss) was used as acquisition software. Imaging analyses were performed by Imaris software v. 7.7 (Bitplane, Inc.). The observed phenotypes were representative of *n* = 100 cells per condition in each experiment.

**Figure 5.**
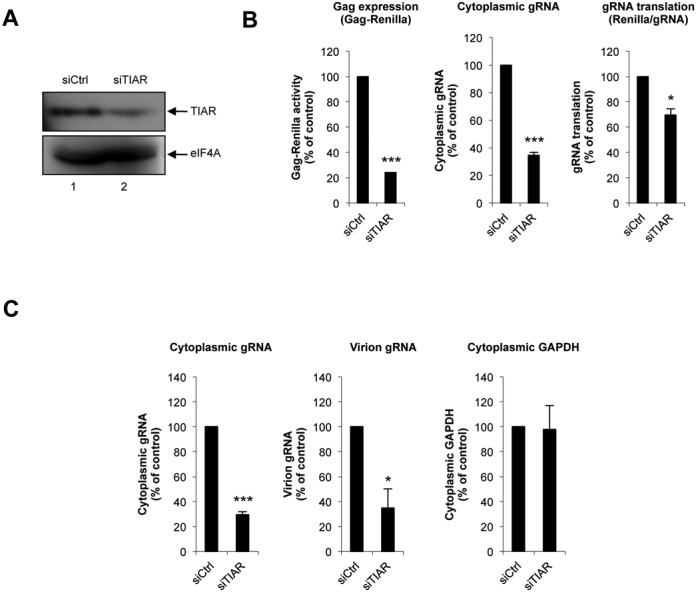
TIAR is required for gene expression from the HIV-2 gRNA. (**A**) Western blot showing knockdown of TIAR in HeLa cells. The translation initiation factor eIF4A was probed using a specific antibody and used as loading control. (**B**) siControl (siCtrl) or siTIAR treated HeLa cells were transfected with the pRod10-*Renilla* provirus (Supplementary Figure S3) for 24 h before analysis of Gag-*Renilla* expression (left panel) and cytoplasmic levels of the gRNA (middle panel). The effect of TIAR knockdown on translation was then determined by normalizing the Gag-*Renilla* activity by the cytoplasmic levels of gRNA and presented in the right panel. Results are expressed as percentage of the control (arbitrary set to 100%) and expressed as mean +/− SD from three independent experiments. (**C**) Control (siCtrl) or siTIAR treated HeLa cells were transfected with the pRod10 full-length provirus for 24 h before analysis of levels of cytoplasmic (middle panel) and virion-associated gRNA (right panel). The cytoplasmic level of GAPDH mRNA was quantified in parallel and used as an internal control (left panel). Results are expressed as percentage of the control (arbitrary set to 100%) and expressed as mean +/− SD from three independent experiments. (* correspond to a *P*-value < 0,05; *** correspond to a *P*-value < 0,001).

**Figure 6.**
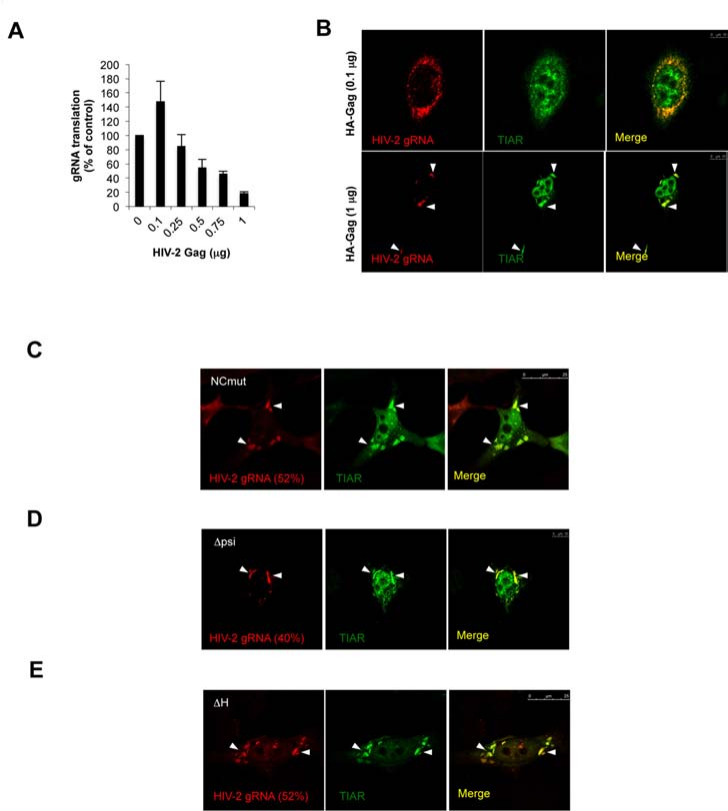
Gag levels control stress granule assembly independently of its binding to the gRNA. (**A**) HeLa cells were co-transfected with the pRod10-*Renilla* vector and increasing amounts (from 0.1 to 1 μg) of the pCIneo-HA-HIV-2 Gag vector (pCIneo-HA-EGFP was used as control). The efficiency of HIV-2 gRNA translation was determined at 24 hpt by measuring the ratio between Gag-*Renilla* expression over the cytoplasmic concentration of the gRNA. Results are expressed as percentage of the control (arbitrary set to 100%) and expressed as mean +/− SD from three independent experiments. (**B**) HeLa cells were co-transfected with 0.5 μg of HIV-2 proviral DNA (pRod10), 0.25 μg of pEGFP-TIAR and 0.1 μg (upper panels) or 1 μg of pCIneo-HA-HIV-2 Gag (lower panel) and subjected to RNA FISH and spectral confocal microscopy as described in Materials and Methods. TH2RNP primarily accumulates in the cytoplasm at low concentration of the transfected Gag vector (upper panels, scale bar 10 μm) and in stress granules at high concentration of Gag as indicated by arrowheads (lower panels, scale bar 10 μm). (**C**) HeLa cells were co-transfected with 0.5 μg of ΔNC HIV-2 proviral DNA (pSVR) and 0.25 μg of pEGFP-TIAR and subjected to RNA FISH and spectral confocal microscopy as described in Materials and Methods. HIV-2 gRNA and TIAR accumulate in cytoplasmic stress granules (scale bar 25 μm). (**D**) HeLa cells were co-transfected with 0.5 μg of Δpsi HIV-2 proviral DNA (pSVR) and 0.25 μg of pEGFP-TIAR and subjected to RNA FISH and spectral confocal microscopy as described in Materials and Methods. HIV-2 gRNA and TIAR accumulate in cytoplasmic stress granules (scale bar 10 μm). (**E**) HeLa cells were co-transfected with 0.5 μg of ΔH HIV-2 proviral DNA (pSVR) and 0.25 μg of pEGFP-TIAR and subjected to RNA FISH and spectral confocal microscopy as described in Materials and Methods. HIV-2 gRNA and TIAR accumulate in cytoplasmic stress granules (scale bar 25 μm). The percentage of each TH2RNP localizing in stress granules is indicated.

**Figure 7.**
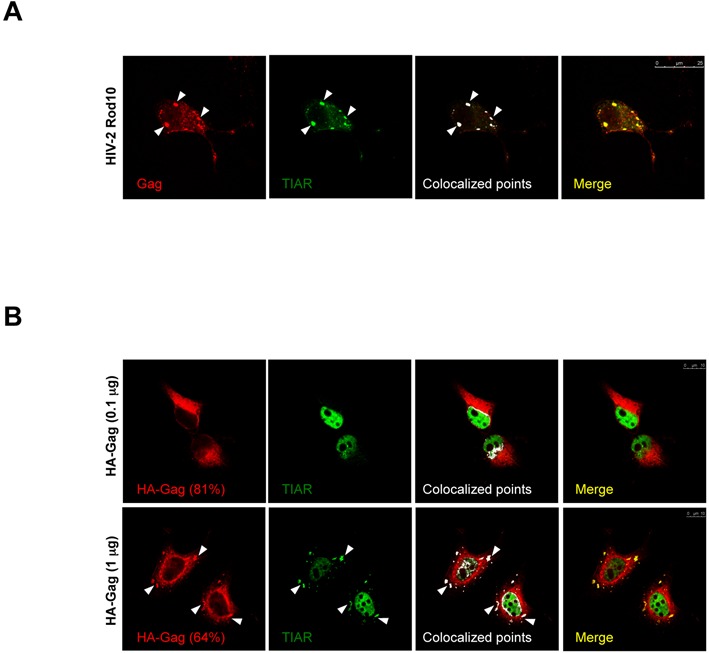
HIV-2 Gag induces and localizes in stress granules. (**A**) HeLa cells were co-transfected with 0.5 μg of HIV-2 proviral DNA (pRod10), 0.25 μg of pEGFP-TIAR and subjected to immunofluorescence (IF) and spectral confocal microscopy as described in Materials and Methods (scale bar 25 μm). (**B**) HeLa cells were co-transfected with 0.1 μg (upper panels) or 1 μg (lower panels) of pCIneo-HA-HIV-2 Gag vector and 0.25 μg of pEGFP-TIAR and subjected to IF and spectral confocal microscopy as described in Materials and Methods (scale bars 10 μm).

## RESULTS

### HIV-2 replication induces the assembly of cytoplasmic granules

Stress granules and P-bodies are involved in the replication cycle of several RNA viruses including Respiratory Syncytial virus, Hepatitis C Virus and Measles Virus (14,15,28–30). As such, we have recently shown that HIV-1 has the ability to inhibit stress granule assembly even upon induced cellular stress suggesting that the formation of these mRNA granules is detrimental to viral replication (14,16,17). In order to determine whether such a property was conserved in the closely related lentivirus HIV-2, we induced eIF2α-dependent or eIF2α-independent assembly of stress granules by treating HIV-1- or HIV-2-expressing HeLa cells with sodium Arsenite or Pateamine A (Figure 1A). Given the difficulties to detect HIV-2 Gag by IF in cells, we also included a previously described HIV-2 Rod-GFP vector in our analysis (31). As expected, drug-treated cells presented the massive aggregation of endogenous G3BP1 and TIAR proteins in stress granules irrespective of eIF2α phosphorylation (Figure 1A, see Arsenite and Pateamine and Supplementary Figure S1). In agreement with our previous results (16), HeLa cells expressing HIV-1 were refractory to stress granule assembly regardless the drug treatment (Figure 1A, see HIV-1 NL4–3). However, as judged by Gag or GFP staining, HIV-2-expressing cells assembled stress granules induced by both drug treatments (Figure 1A and Table 1). These data indicate that the ability to interfere with stress granules assembly observed with HIV-1 is not conserved in the closely related virus HIV-2. More importantly, we observed that a significant fraction of the untreated HIV-2 expressing cells (HIV-2 Rod10 or Rod-GFP, respectively) presented the spontaneous assembly of endogenous TIAR and G3BP1 in stress granules in the absence of any drug-induced cellular stress indicating that HIV-2 expression *per se* induces the assembly of stress granules (Figure 1A, see untreated and Table 1).

**Table 1.** Percentage of HIV-expressing cells presenting stress granule assembly

Virus	Untreated (% cells with SG)	Arsenite^a^ (% cells with SG)	Pateamine A^a^ (% cells with SG)
HIV-1 NL4-3	2	7	7
HIV-2 Rod10	38	74	74
HIV-2 Rod10-GFP	41	74	73

^a^Arsenite and Pateamine A concentrations are indicated in the text.

Given the striking differences observed between HIV-1 and HIV-2 regarding their ability to modulate stress granule assembly, we queried whether HIV-1 was able to block the assembly of stress granules induced by HIV-2 (Figure 1B). Therefore, we co-transfected an HIV-1-NL4–3-RFP molecular clone (32) together with the HIV-2 Rod-GFP vector and analyzed stress granule assembly in untreated and Pateamine A-treated cells by staining the endogenous marker G3BP1 (Figure 1B). Interestingly, we were able to detect assembly of HIV-2- or Pateamine A-induced stress granules only in cells in which HIV-1 was absent (Figure 1B, see untreated or Pateamine A) suggesting that HIV-1 has also the ability to interfere with HIV-2-induced assembly of stress granules.

### HIV-2 induces assembly of *bona fide* stress granules triggered in response to mild levels of eIF2α phosphorylation

Stress granules contain mRNAs stalled at the initiation step of translation and therefore they are enriched in 40S small ribosomal subunits and a subset of translation initiation factors including eIF4G, PABP, eIF3 amongst others (33). However, it has been described that some viruses recruit stress granule components to their replication sites and drive the assembly of stress granules-like structures that usually lack translation initiation factors (14,15,34,35). Thus, in order to determine whether HIV-2-induced stress granules were *bona fide* stress granules enriched in translation initiation factors, we looked for the presence of eIF4GI and eIF3b (Figure 2A). As observed, HIV-2-induced stress granules contained both eIF4GI and eIF3b in addition to TIAR and G3BP1 suggesting that they correspond to *bona fide* stress granules containing stalled translation initiation complexes.

Since stress granule assembly induced by several viral infections usually involves PKR activation and eIF2α phosphorylation (14,15,30,36), we evaluated Ser51 phosphorylation of this translation initiation factor in HIV-2-expressing HeLa cells by IF (Figure 2B). We observed that HIV-2-positive cells presenting stress granules (as judged by the presence of eIF3b, upper panel) presented low but detectable levels of staining for Ser51 phosphorylation suggesting that HIV-2-induced stress granule assembly involves the phosphorylation of eIF2α. However, it should be noted that the level of eIF2α phosphorylation in HIV-2-expressing cells was considerably much lower than that observed upon Arsenite treatment suggesting that HIV-2 expression may not affect cellular protein synthesis through this pathway (Figure 2B, lower panel).

In order to determine whether HIV-2-induced stress granule assembly resulted from disruption of the host translational machinery, we co-transfected the HIV-2 proviral DNA together with a *Renilla* luciferase reporter gene (Figure 2C). As observed, steady-state translation of the reporter gene (*Renilla* activity normalized by cytoplasmic *Renilla* mRNA) was unaffected by HIV-2 expression indicating that despite the induction of stress granule assembly, the host translational machinery was not severely compromised by HIV-2 replication. In agreement with these data, metabolic labeling of HIV-2-expressing cells performed at different time points showed that cellular protein synthesis was not impaired by viral replication (Figure 2D). Together, these data indicate that HIV-2 expression induces the spontaneous assembly of *bona fide* stress granules in response to a mild increase in eIF2α-P suggesting that these cytoplasmic structures could play a role during viral replication.

### The HIV-2 gRNA localizes together with stress granule components

We have previously reported major differences between HIV-1 and HIV-2 expression at the translational level (5). Indeed, our data showed that the presence of RNA structures within the 5′-UTR of the HIV-2 gRNA, and more particularly in the architecture of the TAR stem-loop, was responsible for its poor association with the host translational machinery (5). Thus, we have now investigated further whether the low translational activity of HIV-2 compared to HIV-1 could also reflect differences in the subcellular localization of the gRNA. For this, we have used fluorescent *in situ* hybridization (FISH) coupled to spectral confocal microscopy to analyze whether the HIV-2 gRNA could be present in stress granules. Our imaging analysis revealed that the HIV-2 gRNA presented two main patterns of localization (Figure 3A). In the first localization pattern, we observed the gRNA localizing diffusely in the cytoplasm in 56% of the cells (Figure 3A, left panel). The second localization pattern (44% of the observed cells) showed large cytoplasmic granules that resembled stress granules (Figure 3A, right panel).

To determine whether HIV-2 gRNA granules corresponded to the stress granules characterized above, we looked at the HIV-2 gRNA localization with respect to different stress granule markers. It should be mentioned that for the remaining of the experiments we have used EGFP-tagged versions of the stress granule markers in order to facilitate RNA detection by FISH and confocal microscopy. As we are aware that overexpression of these markers can spontaneously induce stress granule formation (33), we have kept the amount of transfected plasmids to a low level in order to minimize their impact on granule formation. In our hands, only a very minor fraction of cells presented the spontaneous assembly of stress granules due to ectopic expression of the markers (data not shown). Interestingly, the HIV-2 gRNA signals were coincident with that of stress granule markers G3BP1, FXR1 and TIAR (Supplementary Figures S2A, S2B and Figure 3B, respectively). Interestingly, we observed that TIAR also co-localized together with the HIV-2 gRNA when it was present diffuse in the cytoplasm (Figure 3B, 38% of localization in the cytoplasm and 62% localization in stress granules), suggesting the formation of a cytoplasmic TIAR/gRNA complex that we called TH2RNP complex (see below).

Since cellular mRNAs are in equilibrium between polysomes and stress granules, we next investigated whether this was the case for the TH2RNP complex. For this, we looked at the localization of the complex in the presence of cycloheximide and puromycin (Figure 3C and D, respectively). Cycloheximide treatment is known to freeze polysomes resulting in the dissolution of stress granules (33). Inversely, puromycin treatment induces rapid ribosome dissociation resulting in the assembly of enlarged stress granules (33). Consistent with data presented above, we observed that cycloheximide treatment resulted in the complete localization of the TH2RNP complex diffuse in the cytoplasm (Figure 3C) whereas puromycin treatment led to the incorporation of this complex in enlarged stress granules (Figure 3D). Thus, these data suggest that the TH2RNP complex is in equilibrium between polysomes and stress granules.

A characteristic of stress granules is that they are often visualized in close proximity with P-bodies (33). In agreement with this notion, we observed that the TH2RNP present in stress granules localized close to Dcp1-containing P-bodies further confirming that the TH2RNP accumulates in *bona fide* stress granules during viral replication (Figure 3E).

### Assembly of a functional TH2RNP complex requires both the RNA recognition motif and the proline-rich sequence recognition domains of TIAR

The surprising observation that the HIV-2 gRNA was colocalized with TIAR both diffuse in the cytoplasm and in stress granules prompted us to investigate whether TIAR could directly interact with the HIV-2 gRNA. For this, we co-transfected the HIV-2 Rod10 molecular clone together with a HA-TIAR-expressing vector in order to carry out RNA immunoprecipitation and look for the presence of the HIV-2 gRNA (Figure 4A). As observed, HA-TIAR was specifically precipitated with our anti-HA antibody but not with the mouse IgG1 control (Figure 4A, left panel). In addition, the HIV-2 gRNA was detected exclusively from the TIAR associated RNAs but not from the IgG1 control or from reactions in which the RT was omitted confirming the formation of the TH2RNP in cells (Figure 4A, right panel).

Since TIAR is composed of three RNA recognition motifs (RRM) and one Proline-rich sequence recognition domain (PRD), we then asked whether one of these domains could be sufficient to promote assembly of the TH2RNP complex. We first looked at the co-localization of both the RRM and PRD truncated proteins with the HIV-2 gRNA in stress granules. Results presented in Figure 4B show that only the TIAR-RRM fragment was present together with the gRNA in stress granules (upper panel of Figure 4B) whereas the TIAR-PRD was found to disperse in the cytoplasm. This is also consistent with the inability of the PRD to be recruited to stress granules when overexpressed (37). However, neither of these fragments on its own was able to efficiently support HIV-2 replication as indicated by the decrease of RT activity in the cell culture supernatant (Figure 4C). This suggests that both the RRM and PRD motifs are needed to support the formation of a functional TH2RNP.

### TIAR is critical for Gag synthesis from the HIV-2 gRNA

Based on the data presented above, we wanted to examine the role of TIAR during HIV-2 replication. Besides its ability to drive stress granule assembly (38), TIAR has been involved in different post-transcriptional processes including alternative splicing and translational repression of mRNAs containing AU-rich elements (39–43). Thus, to further investigate the impact that TIAR could have on gene expression from the viral transcript we first used a previously described siRNA duplex (44) to reduce TIAR expression from HeLa cells (Figure 5A).

To measure the effects of TIAR knockdown on gene expression from the HIV-2 gRNA, we take advantage of the pRod10-Renilla vector, which contains the *Renilla* luciferase reporter gene inserted in frame within the Gag coding region (5) (Supplementary Figure S3). Transfection of this reporter proviral clone in control (siCtrl) or TIAR-depleted cells (siTIAR) revealed that Gag synthesis was strongly inhibited upon TIAR knockdown (Figure 5B, left panel). Quantification of the gRNA present in the cytoplasmic fraction by RT-qPCR showed that the effect of TIAR depletion on Gag expression could be mostly attributed to a reduction in the level of the gRNA present in the cytoplasm (Figure 5B, middle panel). However, analysis of the translational efficiency (Supplementary Figure S3) revealed that gRNA translation was also significantly diminished upon depletion of TIAR (Figure 5B, right panel). Of note, we observed that the reduction of the cytoplasmic gRNA was not related to an increased release of viral particles as both cytoplasmic and virion-associated gRNAs were similarly reduced by TIAR-depletion (Figure 5C, left and middle panels). Still, this effect seems to be specific for this viral transcript as the level of the cellular GAPDH mRNA remained unaffected in TIAR-depleted cells (Figure 5C, right panel). Moreover, the RT activity detected in the culture supernatant was also diminished in TIAR-depleted cells (data not shown), confirming the negative impact that TIAR knockdown has on viral Gag expression and viral replication. Together, these results strongly indicate that TIAR is a host factor essential in determining the cytoplasmic fate of the HIV-2 gRNA.

### Gag levels control gRNA translation and its assembly in stress granules

Translation of the gRNA is critical to ensure the levels of Gag that are required for packaging and viral particle assembly. However, it is assumed that the binding of Gag to the packaging signal present within the 5′-UTR must segregate the gRNA away from the translational machinery and induce the switch toward the packaging process (1). In fact, previous studies using reporter systems have shown that the Gag protein could modulate its own expression by controlling gRNA translation (8,45). Therefore, we next asked whether the levels of the Gag polyprotein could have an impact on gRNA translation and its accumulation in stress granules. For this, we first transfected the HIV-2 Rod10-Renilla provirus together with increasing amounts of a vector coding its cognate, HA-tagged, Gag protein (Figure 6A). In agreement with what was previously observed using reporter systems (8,45), we observed that expression of Gag *in trans* exerted a bimodal effect on its own expression during viral replication as it first stimulates gRNA translation at low concentration to then inhibit it at higher concentrations (compare 0.1 μg and 1 μg of transfected vector). We then analyzed the localization of the TH2RNP complex under low (0.1 μg) and high levels (1 μg) of transfected HA-Gag vector (Figure 6B). Interestingly, we observed that expression of low levels of Gag *in trans* was accompanied by a preferential localization of the gRNA and TIAR diffuse in the cytoplasm (Figure 6B, upper panels). This contrasts with the effects of high levels of Gag, which induced the massive accumulation of the gRNA and TIAR in stress granules (80% of cells presenting the TH2RNP in stress granules) (Figure 6B, lower panels). These results not only confirm that the TH2RNP accumulates in stress granules when active translation is impaired (Figure 3D), but they also suggest that the levels of the Gag polyprotein could play a role in controlling both translation and the cytoplasmic localization of the TH2RNP complex.

Then, to further address any potential role of the Gag/gRNA interaction on the assembly of the gRNA in stress granules, we used three previously described HIV-2 Rod10-derived proviral clones in which the interaction of Gag with the gRNA has been disrupted (12). The first construct contains mutations in the NC zinc fingers, which abolishes the Gag/gRNA interaction (NCmut in Figure 6C). The second clone harbors deletions in the major packaging signal, which abolishes encapsidation by Gag (Δpsi in Figure 6D). The third one possesses several stop codons within the Gag coding region and thus, although the gRNA is engaged in translation, Gag synthesis is prematurely arrested (ΔH in Figure 6E). To most of our surprise, none of these mutations altered the localization of the TH2RNP complex in stress granules indicating that assembly of the gRNA in stress granules does not rely on any interaction with Gag or the presence of Gag but rather depends on its translational status.

### HIV-2 Gag accumulates in stress granules in the absence of a ‘packageable’ gRNA

Results presented above indicate that the Gag/gRNA interaction is not directly involved in the accumulation of the gRNA in stress granules despite the fact that expression *in trans* of high levels of Gag are able to repress gRNA translation and induce its assembly in stress granules. Thus, we reasoned that if the TH2RNP assembled in stress granules corresponds to a post-translation intermediate occurring prior gRNA packaging, the Gag protein should also be able to accumulate in stress granules in order to encounter the gRNA and initiate the packaging process. To test this hypothesis, we first looked whether the Gag protein expressed in *cis* from the HIV-2 Rod10 molecular clone could be detected in stress granules (Figure 7A). As observed, some of the HIV-2 Gag present in the cytoplasm co-localized with TIAR in stress granules, indicating that the viral protein also accumulates in stress granules during viral replication.

Finally, we asked whether the localization of Gag in stress granules could be dependent on the presence of the gRNA. For this, we analyzed whether HIV-2 Gag synthesized from the transfected plasmid could also be accumulated in stress granules (Figure 7B). It should be mentioned that this vector only contains the Gag coding region and thus it lacks any packaging signal present within the 5′-UTR of the gRNA. Interestingly, we observed that expression of HIV-2 Gag not only induced the re-localization of TIAR from the nucleus to stress granules but we also observed that HIV-2 Gag accumulated with TIAR in such cytoplasmic structures in the absence of any packageable gRNA. Of note, Gag-induced stress granules were dependent on the levels of ectopically expressed protein as low levels of Gag (as judged by the amount of transfected DNA) corresponded to a localization of most of the TIAR protein in the cell nucleus with Gag localizing in the cytoplasm (81% of observed cells) whereas high levels of Gag were associated to the presence of TIAR and Gag in stress granules (64% of observed cells). Taken together, these data indicate that HIV-2 Gag has the ability to induce the assembly of stress granules and to accumulate in these cytoplasmic structures in response of its cytoplasmic levels but independently from its interaction with the gRNA.

Interestingly, a recent report shows that HIV-1 Gag is the major determinant for the blockade of stress granule assembly imposed by HIV-1 (17). Therefore, this highlights major differences between the two human viruses in their subcellular localization and the role of the Gag polyprotein in this process.

## DISCUSSION

HIV-2 is less pathogenic than HIV-1 as it is better controlled by the immune system (46) and possesses a replication fitness much lower than that of HIV-1 (47,48). However, both viruses have the same pathogenic potential (49) and the same ability to infect and establish the proviral state (48). Our previous results have proposed that the poor association of the HIV-2 gRNA with the host translational machinery partially accounts for these differences (5). Data presented here show that HIV-2 expression induces the spontaneous assembly of *bona fide* stress granules containing classical markers such as G3BP1, TIAR and translation initiation factors eIF4GI and eIF3 (Figures 1 and 2). This is in sharp contrast with HIV-1, which has the ability to impair stress granule assembly even upon induced cellular stress (Figure 1) (16,17). Interestingly, we also show that HIV-1 has the ability to interfere with HIV-2-induced stress granule assembly in cells expressing both viruses (Figure 1), suggesting that modulation of stress granule assembly could account for the differences in replication between both viruses.

Although assembly of HIV-2-induced stress granules was associated to a low level of phosphorylation of eIF2α, it was not accompanied by a detectable impairment of the host cell translational machinery (Figure 2). These data suggest that assembly of stress granules in HIV-2 expressing cells might be an important step during viral replication occurring without disrupting the cellular homeostasis. Indeed, we observed the full-length unspliced HIV-2 gRNA localizing with several core components of stress granules including TIAR, G3BP1 and FXR1 (Figure 3 and Supplementary Figure S2). This prompted us to look for a role of TIAR in regulating gene expression from this viral transcript. This was carried out by performing a partial knockdown of TIAR in HIV-2 expressing cells that resulted in a strong decrease of the cytoplasmic concentration of the gRNA (Figure 5). Interestingly, this decrease was also accompanied by a slight impairment of its translational efficiency, the combination of both mechanisms having a drastic effect on the synthesis of the Gag protein and the resulting production of viral particles (Figure 5 and data not shown). These data indicate that TIAR is an important factor involved in the post-transcriptional control of HIV-2 gene expression. Indeed, we established that assembly of the gRNA in stress granules was associated with a low translational efficiency status (Figures 3 and 6).

From these data, it could be envisioned that stress granules could be used as sites for gRNA storage waiting for the threshold levels of Gag required to initiate the packaging process. This idea is strongly supported by previous work showing that (i) HIV-2 gRNA translation is inefficient compared to HIV-1 (5); (ii) Gag levels are limiting during HIV-2 replication (12) and (iii) compared to HIV-1, cytoplasmic levels HIV-2 gRNA are more stable (50). Moreover, this notion is also in good agreement with the fact that packaging signals are present in all HIV-2 transcripts (12). As such, the accumulation of the HIV-2 gRNA in stress granules could contribute to its selective packaging over the other spliced viral transcripts.

**Figure 8.**
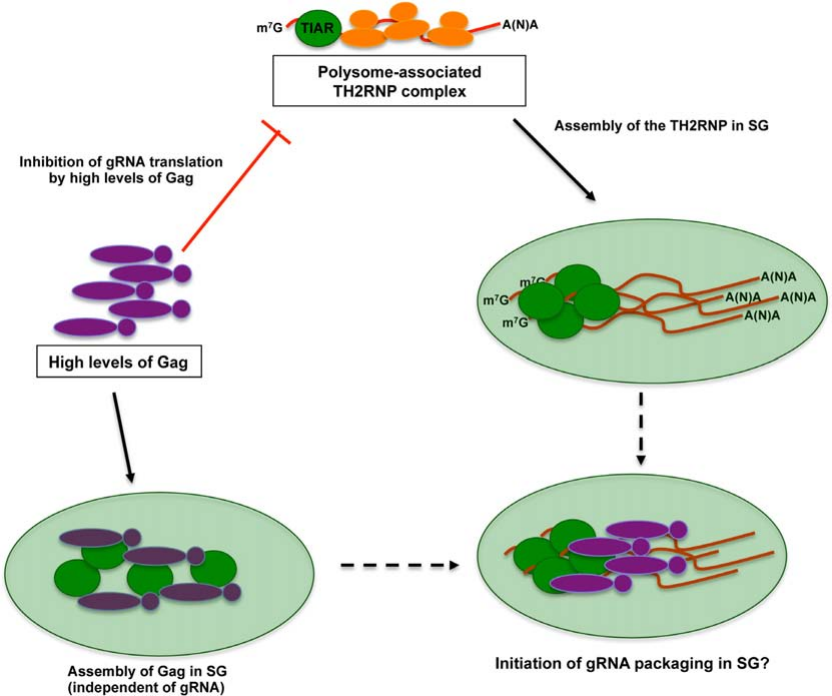
A model for the localization of the TH2RNP complex in stress granules. During active translation, the TH2RNP complex associates with polysomes diffusely in the cytoplasm. Once Gag reaches threshold levels, it inhibits gRNA translation resulting in the accumulation of TH2RNP complex in stress granules independently of its binding to the gRNA. However, high levels of Gag also result in the accumulation of the protein in stress granules independently of the presence of a packageable gRNA. The ability of the gRNA and Gag to accumulate in stress granules suggests that the switch from translation to packaging could occur at these cytoplasmic foci.

Although high levels of Gag expressed in *trans* resulted in the inhibition of gRNA translation and its assembly in stress granules (Figure 6), we were mostly surprised to observe that the assembly of the TH2RNP complex in stress granules also occurred both in the complete absence of Gag or when the Gag/gRNA interaction was disrupted (Figure 6). Nevertheless, this observation could be explained by (i) the low translational efficiency reported for the HIV-2 gRNA (5), which may result in a poor polysome association and (ii) the association of the gRNA with the stress granule assembly factor TIAR that may favor its storage in stress granules. Then, when the Gag polyprotein reaches threshold levels it may encounter the gRNA in stress granules. Consistent with this idea, our data also indicate that the expression of the Gag protein in the absence of a ‘packageable’ gRNA induces the assembly and localizes in stress granules (Figure 7). Thus, it is conceivable to speculate that stress granules may serve as sites of concentration of both the gRNA and the Gag polyprotein that may favor the selective packaging of the HIV-2 genome (Figure 8). Interestingly, it was recently reported that the co-translational RNA packaging mechanism previously described (12) was not the main mechanism by which HIV-2 Gag selects the gRNA for encapsidation but rather this occurs by a *trans* packaging process (51). As such, the authors proposed that HIV-2 Gag primarily package the dimeric form of the gRNA prior encapsidation (51). These observations are in complete agreement with our data and further suggest that Gag and the gRNA may meet each other in stress granules.

Compartmentalization of the gRNA through the assembly of large cytoplasmic mRNPs may be a common feature of viruses that use the same RNA molecule for translation and packaging. Indeed, it is noteworthy that assembly of Ty1 and Ty3 retroelement ribonucleoprotein complexes takes place in yeast P-bodies (52,53). Moreover, the Line-1 retroelement RNA and proteins were shown to accumulate in large cytoplasmic foci together with several stress granule components (54–56) further supporting the idea that transition between translation and packaging of some retroviruses could be compartmentalized. Thus, assembly of the TH2RNP and Gag in stress granules could mimic the role proposed for the SH1RNP assembled in HIV-1 expressing cells (14,16). Interestingly, while this manuscript was in preparation, Valiente-Echeverria *et al.* showed that the Gag protein is the major determinant responsible for the blockade of stress granule assembly in HIV-1-expressing cells (17). These data combined with ours reveal interesting opposite roles of the Gag protein of both viruses in stress granule assembly and further highlight major differences between the two closely related human viruses.

Taken together, our results highlight a novel step during the HIV-2 replication cycle (Figure 8), which involves the accumulation of the gRNA and the Gag protein in stress granules. This process is determined by (i) the formation of the TH2RNP, (ii) the level of translation of the gRNA and (iii) threshold levels of Gag. These data further suggest that the assembly of stress granules in HIV-2 expressing cells could serve as sites where transition from translation to packaging takes place.

## SUPPLEMENTARY DATA

Supplementary Data are available at NAR Online.

SUPPLEMENTARY DATA
