# Supplementary material for: HIV-2 genomic RNA accumulates in stress granules in the absence of active translation
Source: Nucleic Acids Res. 2014 Oct 28;42(20):12861–75. doi: 10.1093/nar/gku1017 (PMC4227750; doi:10.1093/nar/gku1017)
Supplement: SUPPLEMENTARY DATA [file supp_42_20_12861__index.html]

HIV-2 genomic RNA accumulates in stress granules in the absence of active translation — HIV-2 genomic RNA accumulates in stress granules in the absence of active translation — SUPPLEMENTARY DATA 

# HIV-2 genomic RNA accumulates in stress granules in the absence of active translation

## SUPPLEMENTARY DATA

**Files in this Data Supplement:**

- SUPPLEMENTARY DATA
