# Supplementary material for: HIV-2 genomic RNA accumulates in stress granules in the absence of active translation
Source: Nucleic Acids Res. 2014 Oct 28;42(20):12861–75. doi: 10.1093/nar/gku1017 (PMC4227750; doi:10.1093/nar/gku1017)
Supplement: SUPPLEMENTARY DATA [file supp_gku1017_nar-01386-y-2014-File011.pdf]

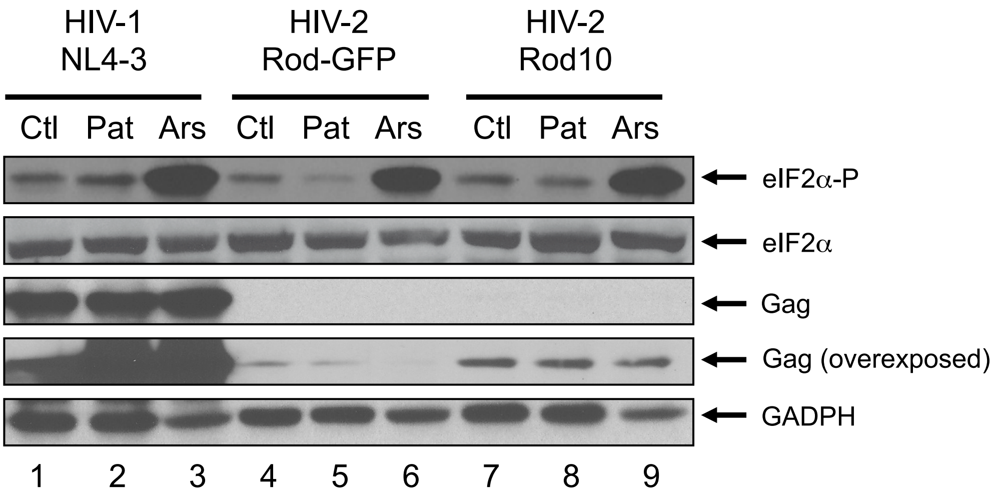

Figure S1: Cell lysates from HIV-1- or HIV-2-expressing cells were analyzed for eIF2α-P (Ser51), eIF2α, Gag and GAPDH by Western blot.
Ctl: Control (Untreated); Pat: Pateamine A; Ars: Sodium Arsenite

A

B

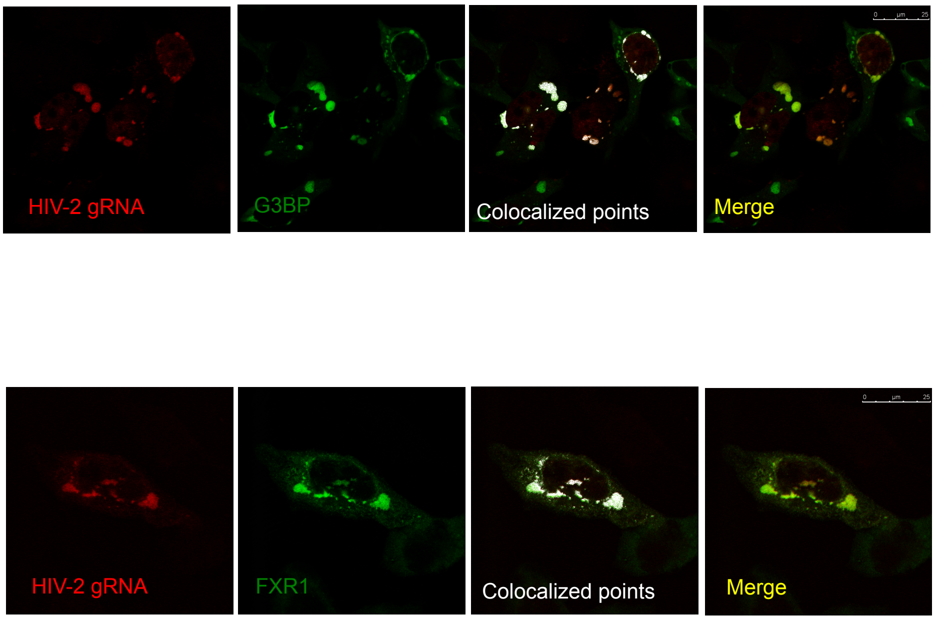

Figure S2: HeLa cells were co-transfected with 0,5 μg of HIV-2 proviral DNA (pRod10) and 0,5 μg of pEGFP-G3BP1 (**A**) or 0,25 μg of EGFP-FXR1 (**B**) and subjected to RNA FISH and spectral confocal microscopy as described in Materials and Methods.

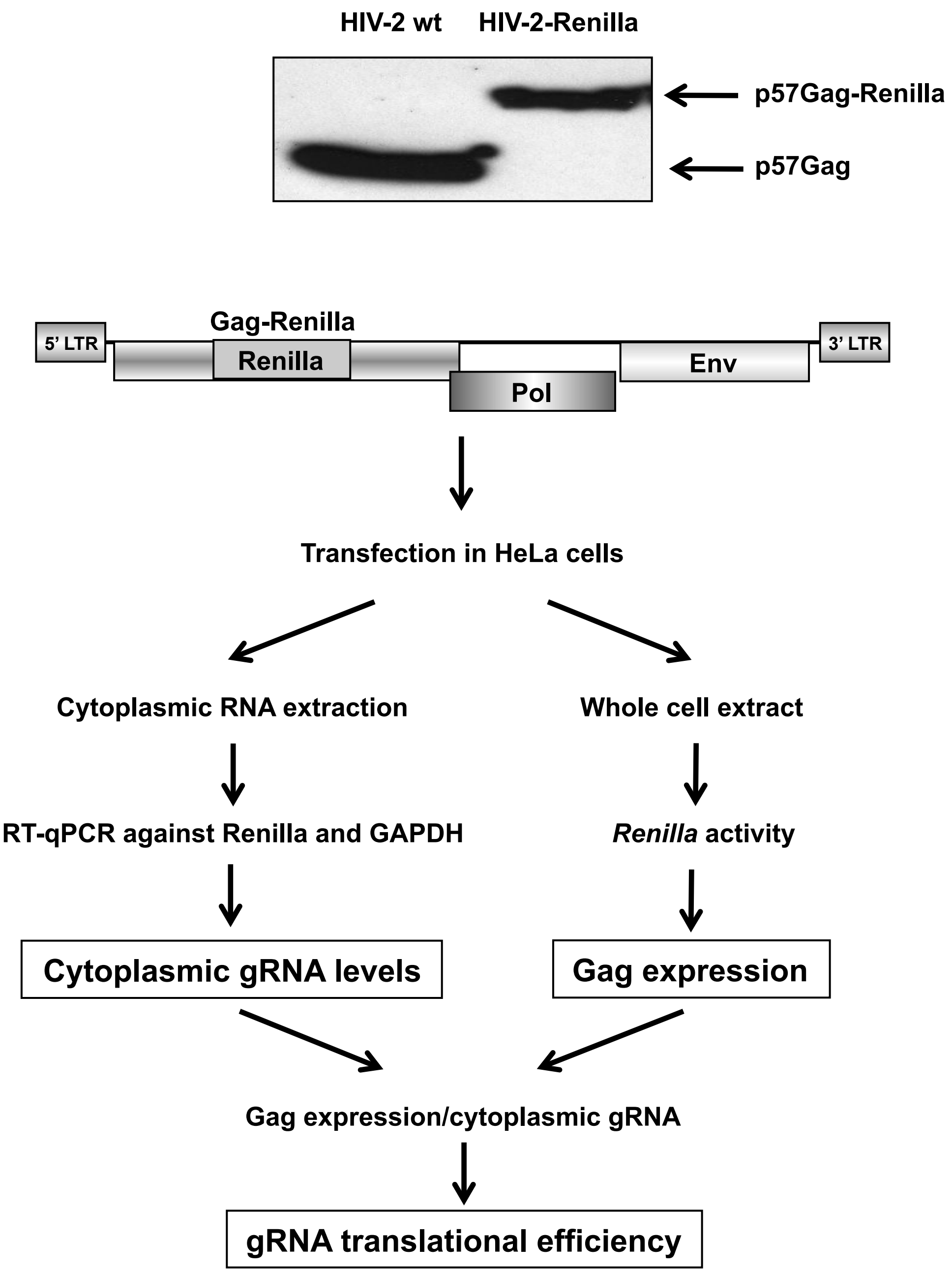

Figure S3: The HIV-2 Rod-*Renilla* proviral DNA produces a fusion Gag-*Renilla* protein (see Western blot). Upon transfection, cell extracts are prepared to both quantify the cytoplasmic levels of the gRNA by RT-qPCR or to measure the activity of the Gag-*Renilla* fusion protein and determine Gag expression. The ratio between Gag expression (Gag-*Renilla* activity) and cytoplasmic gRNA give us the translational efficiency of the gRNA.
